# A method exploiting direct communication between phasor measurement units for power system wide-area protection and control algorithms

**DOI:** 10.1016/j.mex.2017.10.002

**Published:** 2017-10-14

**Authors:** Muhammad Shoaib Almas, Luigi Vanfretti

**Affiliations:** SmarTS Lab, KTH Royal Institute of Technology, Stockholm, Sweden

**Keywords:** Adapting direct relay-to-relay communication for Phasor Measurement Units, IEEE C37.118, Phasor Measurement Unit (PMU), Phasor Data Concentrator (PDC), Smart grid, Synchrophasors, Wide Area Monitoring Protection and Control (WAMPAC)

## Abstract

Synchrophasor measurements from Phasor Measurement Units (PMUs) are the primary sensors used to deploy Wide-Area Monitoring, Protection and Control (WAMPAC) systems. PMUs stream out synchrophasor measurements through the IEEE C37.118.2 protocol using TCP/IP or UDP/IP. The proposed method establishes a direct communication between two PMUs, thus eliminating the requirement of an intermediate phasor data concentrator, data mediator and/or protocol parser and thereby ensuring minimum communication latency without considering communication link delays. This method allows utilizing synchrophasor measurements internally in a PMU to deploy custom protection and control algorithms. These algorithms are deployed using protection logic equations which are supported by all the PMU vendors. Moreover, this method reduces overall equipment cost as the algorithms execute internally in a PMU and therefore does not require any additional controller for their deployment. The proposed method can be utilized for fast prototyping of wide-area measurements based protection and control applications. The proposed method is tested by coupling commercial PMUs as Hardware-in-the-Loop (HIL) with Opal-RT’s eMEGAsim Real-Time Simulator (RTS). As illustrative example, anti-islanding protection application is deployed using proposed method and its performance is assessed. The essential points in the method are:

• Bypassing intermediate phasor data concentrator or protocol parsers as the synchrophasors are communicated directly between the PMUs (minimizes communication delays).
• Wide Area Protection and Control Algorithm is deployed using logic equations in the client PMU, therefore eliminating the requirement for an external hardware controller (cost curtailment)
• Effortless means to exploit PMU measurements in an environment familiar to protection engineers.

## Graphical abstract

**Figure:**
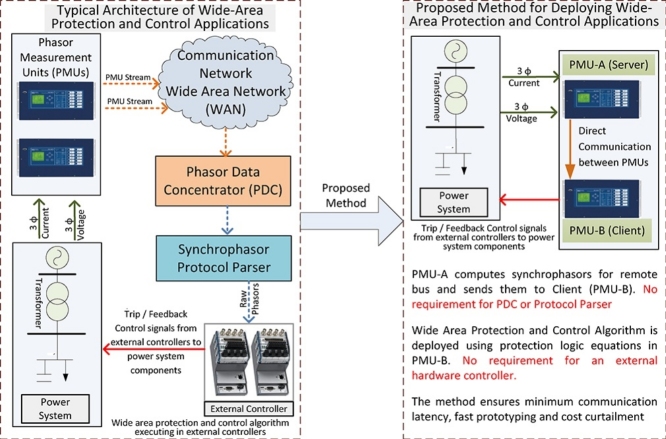

## Method details

For the specific case of synchrophasor-based Wide Area Protection and Control (WAP&C) applications, synchrophasors from two Phasor Measurement Units (PMUs) may be sufficient to perform protection and control actions [1], [2]. This method proposes the use of direct communication between two PMUs to deploy WAP&C applications. This eliminates the requirement of an intermediate Phasor Data Concentrator (PDC) and protocol parser, thus avoiding the non-deterministic delays incurred by these processes. This method utilizes both local and remote synchrophasor measurements internally in a PMU to execute WAP&C algorithms. These algorithms are deployed using protection logic equations [3], which are supported by most PMU vendors. The outputs of the algorithms (e.g. trip or control command) are mapped to the outputs and front panel of the PMU. As these WAP&C utilizes resources of the PMUs, their deployment does not require any additional external hardware controller. Thus the overall time for WAP&C application prototyping is reduced and its deployment cost is curtailed. This method is based on direct relay-to-relay communication that is used in the field of power system protection for achieving fast tripping, deploying tele-protection schemes and for monitoring status of remote protection relays [4]. The authors, in this method, propose using a similar method for PMUs to deploy protection and control applications.

## Explanation of methodology

PMUs operate in a server mode, i.e. they allow a client e.g. a PDC or an application executing on a workstation to connect to it (i.e. client). Once the connection is established between the source (PMU) and the destination (PDC/Workstation), the PMU streams synchrophasor measurements in IEEE C37.118.2 format using TCP/IP or UDP/IP [5]. The proposed method requires modifying this approach, using n-1 PMUs as servers, and 1 PMU that operates in a client mode where the protection or control application can be deployed. The 4 major steps of this method are described below.

### Step 1: Configuring one PMU as a client for the other PMU

**Fig. 1:**
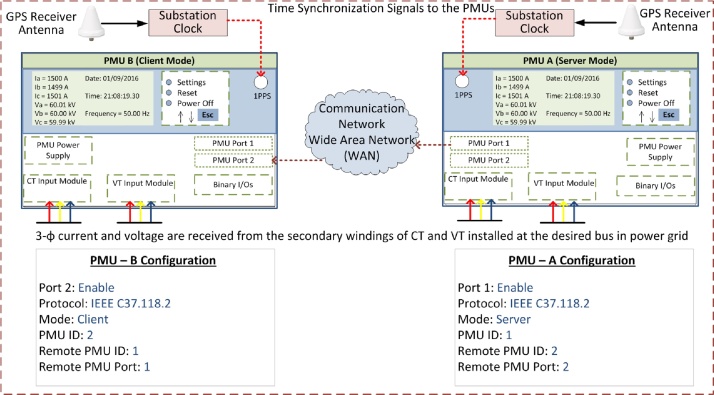
Server-client configurations used in the proposed method.

Hardware PMUs have several communication ports that can be configured to receive or transmit data through a specific communication protocol. As the first step of this method, one of the two PMUs is configured as a “Client” for the other PMU and (itself) operates in the usual “Server” mode. For illustrative purposes, consider two PMUs as PMU-A and PMU-B. PMU-B is configured as a client for PMU-A. Thus PMU-B expects to receive synchrophasors from PMU-A on a particular port at a configured data rate. The required configuration of the two PMUs is shown in Fig. 1.

### Step 2: Mapping remote synchrophasors to local variables in the client PMU

**Fig. 2:**
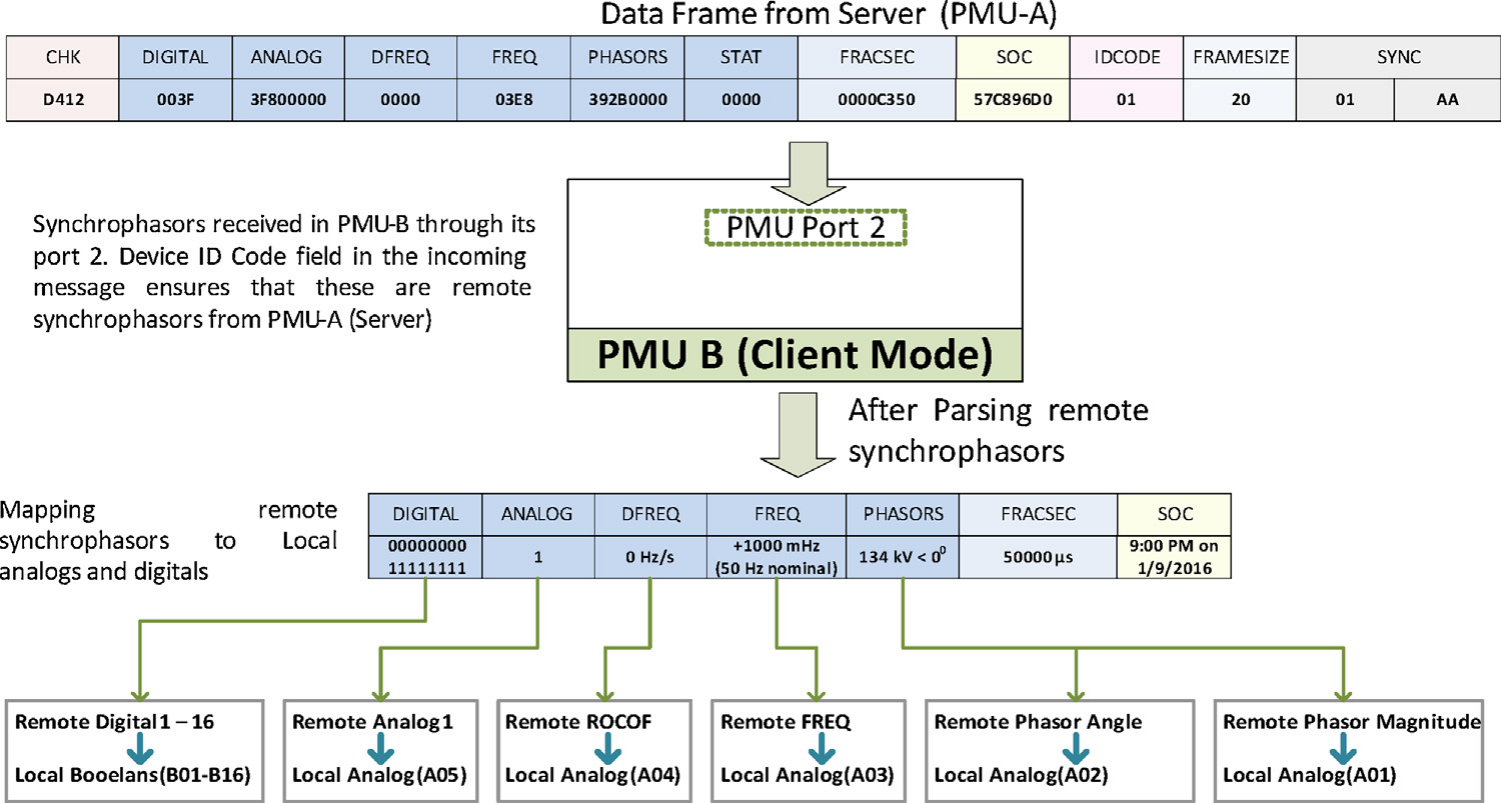
Mapping remote synchrophasors to local analog and digital variables.

Once the communication is established, PMU-B (client) starts receiving and parsing synchrophasor measurements from PMU-A (Server). The remote synchrophasors are parsed as magnitude-angle pairs or real-imaginary pairs as configured in the server (PMU-A). The next step is to map these remote synchrophasor measurements as local variables in PMU-B (Client). Commercial PMUs allow configuring different analog and digital variables to custom values. Each remote synchrophasor is mapped to two local analog variables i.e. one for synchrophasor’s magnitude/real part and other for synchrophasor’s angle/imaginary part. Similarly the remote synchrophasor frequency/ROCOF and Booleans are mapped to local analog and local digital variables respectively. This step is shown in Fig. 2.

### Step 3: Deploying WAP&C algorithms in PMU using protection logic equations

**Fig. 3:**
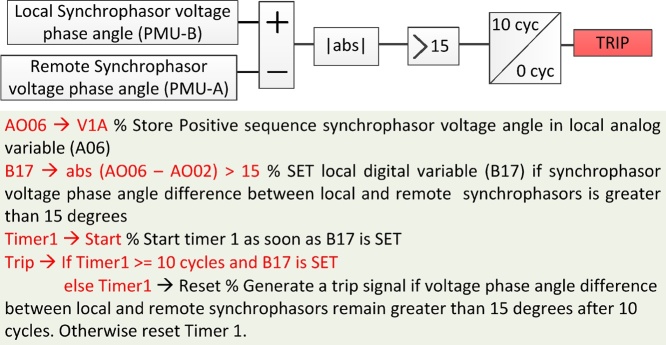
Islanding detection algorithm exploiting local and remote synchrophasors and deployed using protection logic equations.

Once the remote synchrophasors are mapped to local analog and digital variables, custom WAP&C algorithms are deployed using protection logic equations [3]. Several PMU vendors support protection logic equations for protection application flexibility and as a platform for substation automation. With protection logic equations, arithmetic operations can be performed on local and remote synchrophasor signals. An example of a custom algorithm is shown in Fig. 3, which generates a trip signal if the voltage phase angle difference between local (PMU-B) and remote (PMU-A) synchrophasors is greater than 15° for more than 10 cycles. This is a typical case of detecting islanding condition, a situation when a local generator gets disconnected from the rest of the power system [1].

### Step 4: Mapping the outputs of algorithms to trip signals and front panel LEDs of PMU

**Fig. 4:**
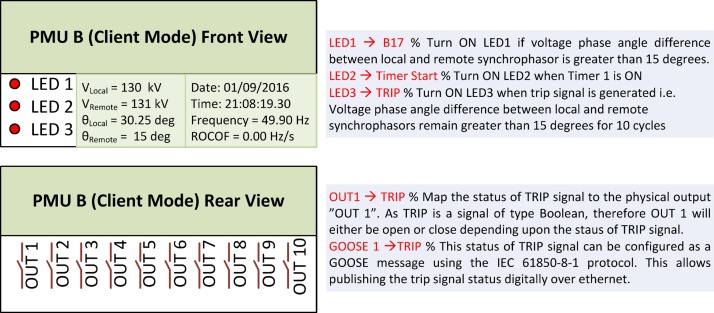
Mapping algorithm important states to the PMU’s front panel LEDs, physical outputs and GOOSE messages.

As the last step of this method, the important outputs of the algorithms, which are trip signals or control commands, are mapped to the physical output of the PMU so that they can be accessed through PMUs’ digital inputs/outputs (I/Os). Furthermore, these important algorithm outputs are mapped to the front panel LEDs of the PMU to give an instant indication of the alarm situation and operational state of the algorithm executed. It is also possible to map the output of the algorithm as IEC 61850-8-1 GOOSE message [6], [7]. This step is shown in Fig. 4.

## Method illustration and validation

The method described in the previous section is illustrated and validated by coupling commercial PMUs from Schweitzer Engineering Laboratories (SEL) [8] as Hardware-in-the-Loop (HIL) with Opal-RT’s eMEGAsim Real-Time Simulator (RTS) [9] and analysing an anti-islanding protection scheme [1]. This type of simulation is termed as Real-Time Hardware-in-the-Loop (RT-HIL) simulation [10].

**Fig. 5:**
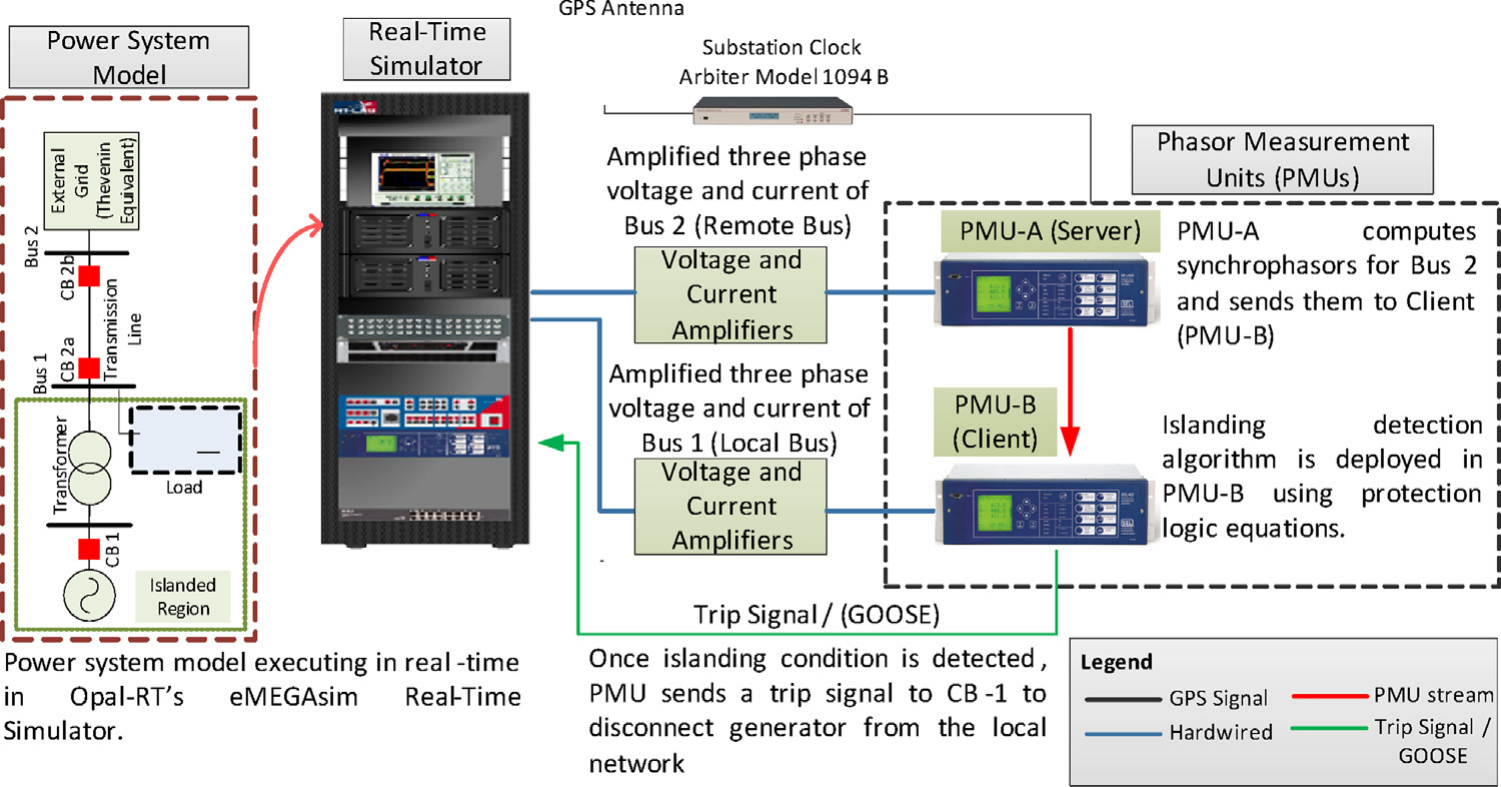
RT-HIL experimental setup for method validation.

**Fig. 6:**
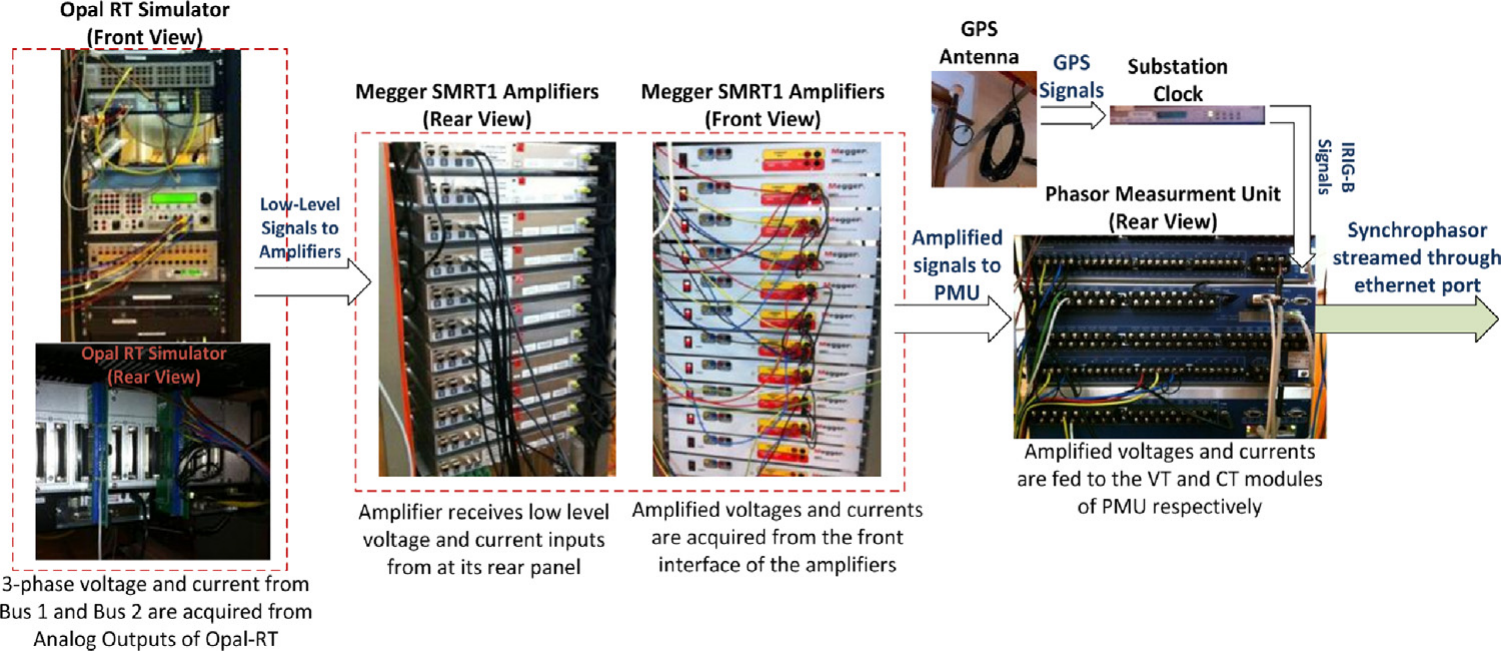
Interface between various hardware components of the RT-HIL experimental setup.

The experimental setup used herein is shown in Fig. 5, and was deployed at the Smart Transmission System Laboratory (SmarTS-Lab) at KTH-Royal Institute of Technology [11]. To perform RT-HIL simulations, a power system consisting of a single generator with its local load and connected to external grid through a transmission line was modelled in MATLAB/Simulink using SimPowerSytems Library and compiled for execution in the RT target. If circuit breakers CB-2a and CB-2b are opened following a fault, the generator together with its local load becomes an island. The standard for “Interconnecting Distributed Resources with Electric Power Systems, IEEE Std. 1547-2008” states that the distributed generator (DG) must be disconnected from the isolated grid within 2 s after an unintentional islanding event [12]. This islanding condition can be identified by utilizing synchrophasors from local bus (Bus 1) and remote bus (Bus 2). As shown in Fig. 5, the three phase currents and voltages of Bus 2 and Bus 1 are fed, after amplification, to the CT and VT inputs of PMU-A (remote) and PMU-B (Local), respectively. The three phase voltage and current signals acquired from RTS’ analog outputs are low-level signals (±10 V and ±20 mA). In order to make these low-level signals compatible with the PMUs CT/VT input (generally 100 V and 1 A), they are amplified using linear amplifiers [13]. PMU-A and PMU-B compute synchrophasor for voltages and currents at Bus 2 (remote) and Bus 1 (Local) respectively. PMU-B is configured as a Client for PMU-A and performs the following functions: it receives synchrophasors from PMU-A (step 1), parses its synchrophasor stream and maps remote synchrophasor to its local analog and digital variables (step 2), executes islanding detection algorithm using local and remote synchrophasors (step 3), and finally, generates trip signals for anti-islanding and indicates alarms on its front panel LEDs (step 4). The hardware interfacing between different components of the experimental setup is shown in Fig. 6.

**Fig. 7:**
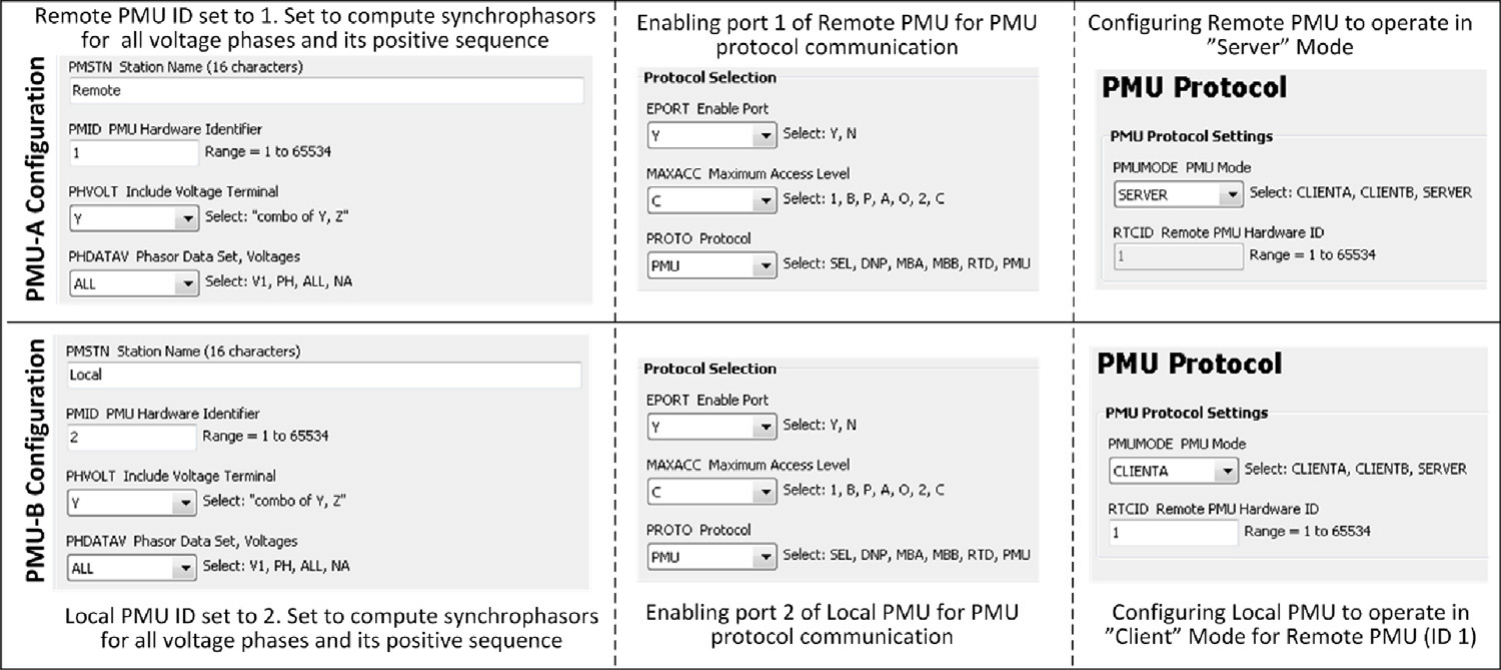
Configuring PMU-B as a client for PMU-A to receive remote synchrophasors. Realization of step 1 of the proposed method.

**Fig. 8:**
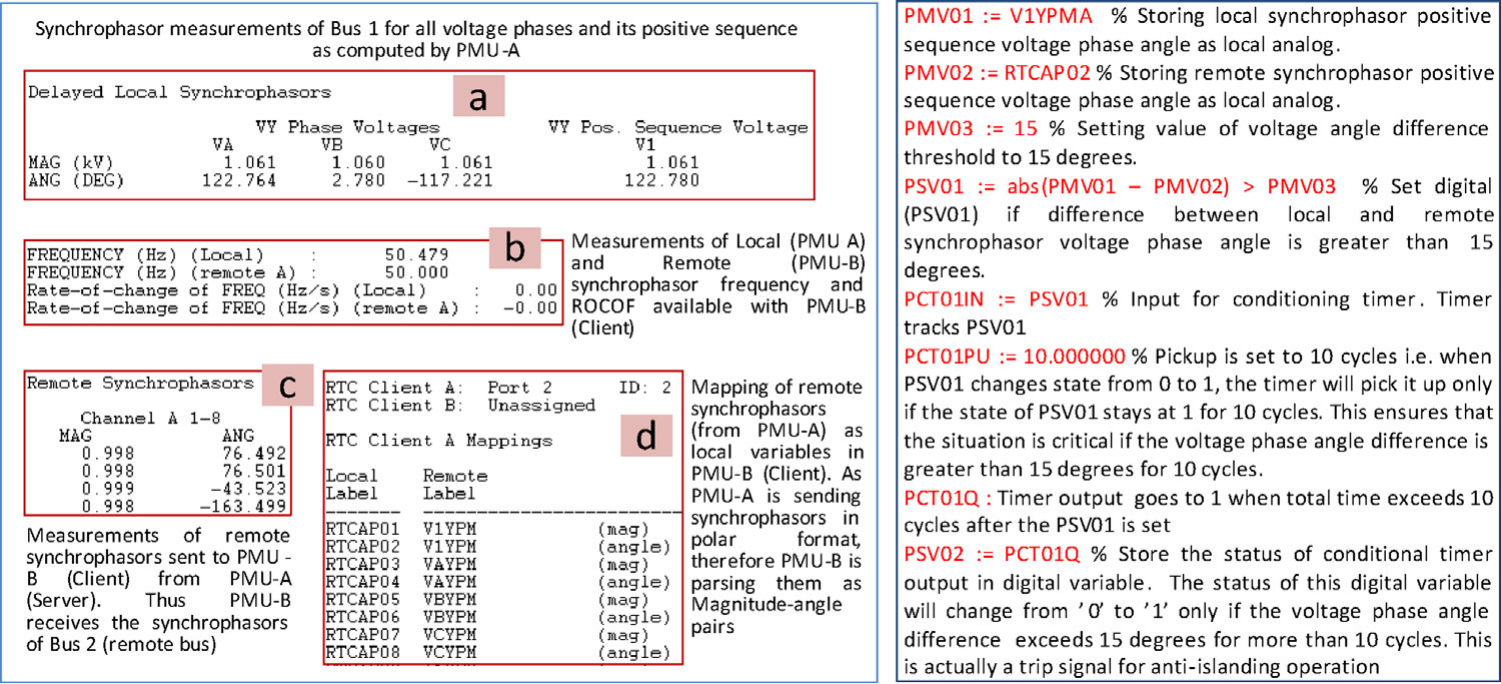
(Left) Screenshot of local computed by PMU-B (a), remote synchrophasors computed by PMU-A and received in PMU-B (b, c), mapping of remote synchrophasors as local variables in PMU-B (d). (Right) Logic equations used to deploy anti-islanding protection algorithm within PMU-B utilizing both local and remote synchrophasors.

**Fig. 9:**
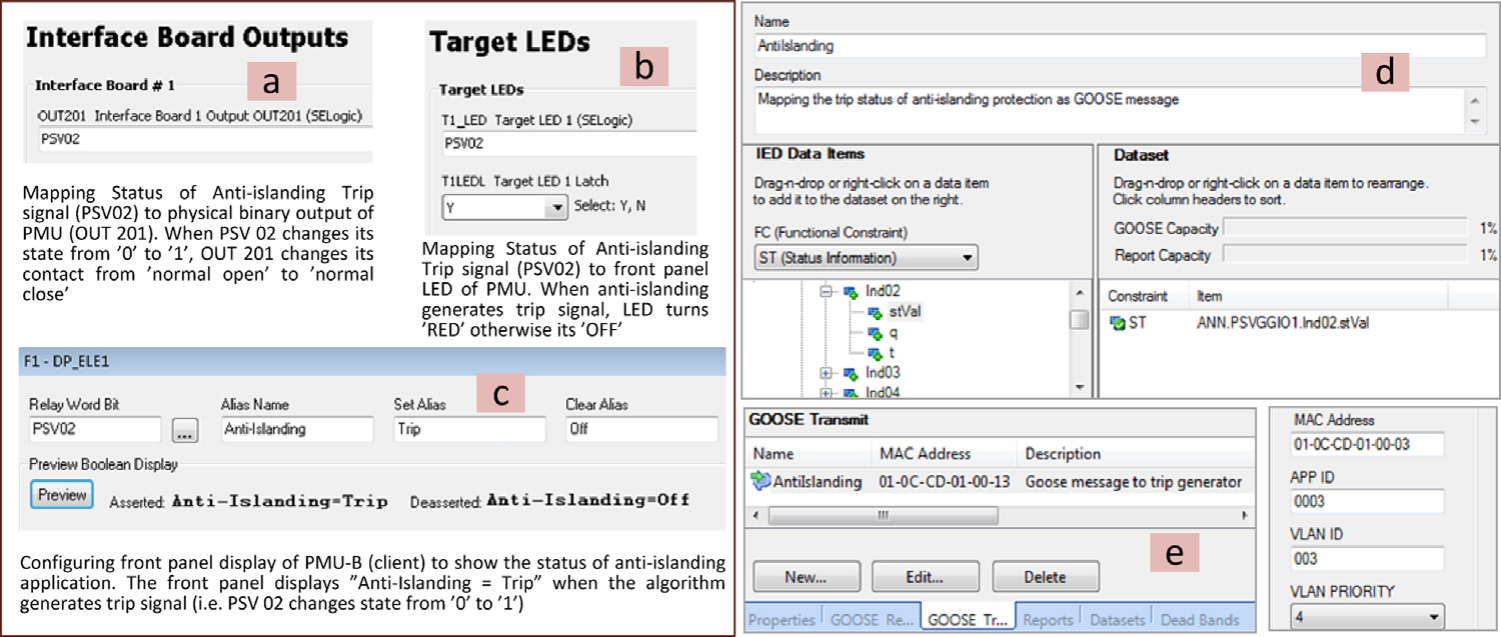
PMU-B configurations to map the important statuses/alarms and trip signals from anti-islanding algorithm execution to (a) physical output, (b) Front panel LED and (c) HMI of PMU, (d) mapping status PSV02 (anti-islanding trip signal) to GOOSE message, and (e) transmitting this GOOSE message through Ethernet port of the PMU.

**Fig. 10:**
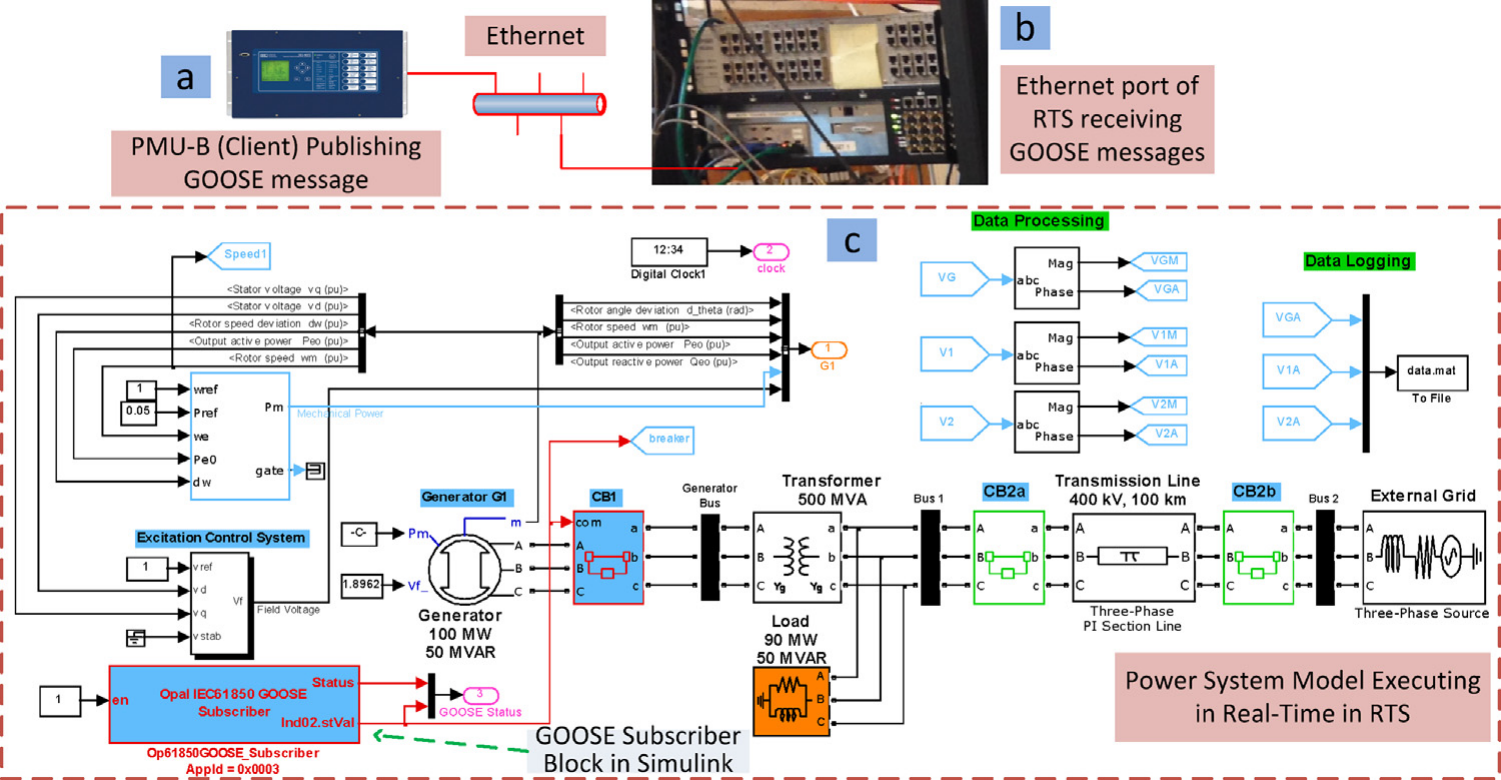
GOOSE message data-flow. (a) Source publishing GOOSE message i.e. PMU-B, (b) receiving this GOOSE message in RTS through its Ethernet port, (c) parsing the GOOSE message in Simulink environment using ‘GOOSE Subscriber’ block. The value of GOOSE message is used to open the circuit breaker CB-1 to execute anti-islanding protection scheme.

**Fig. 11:**
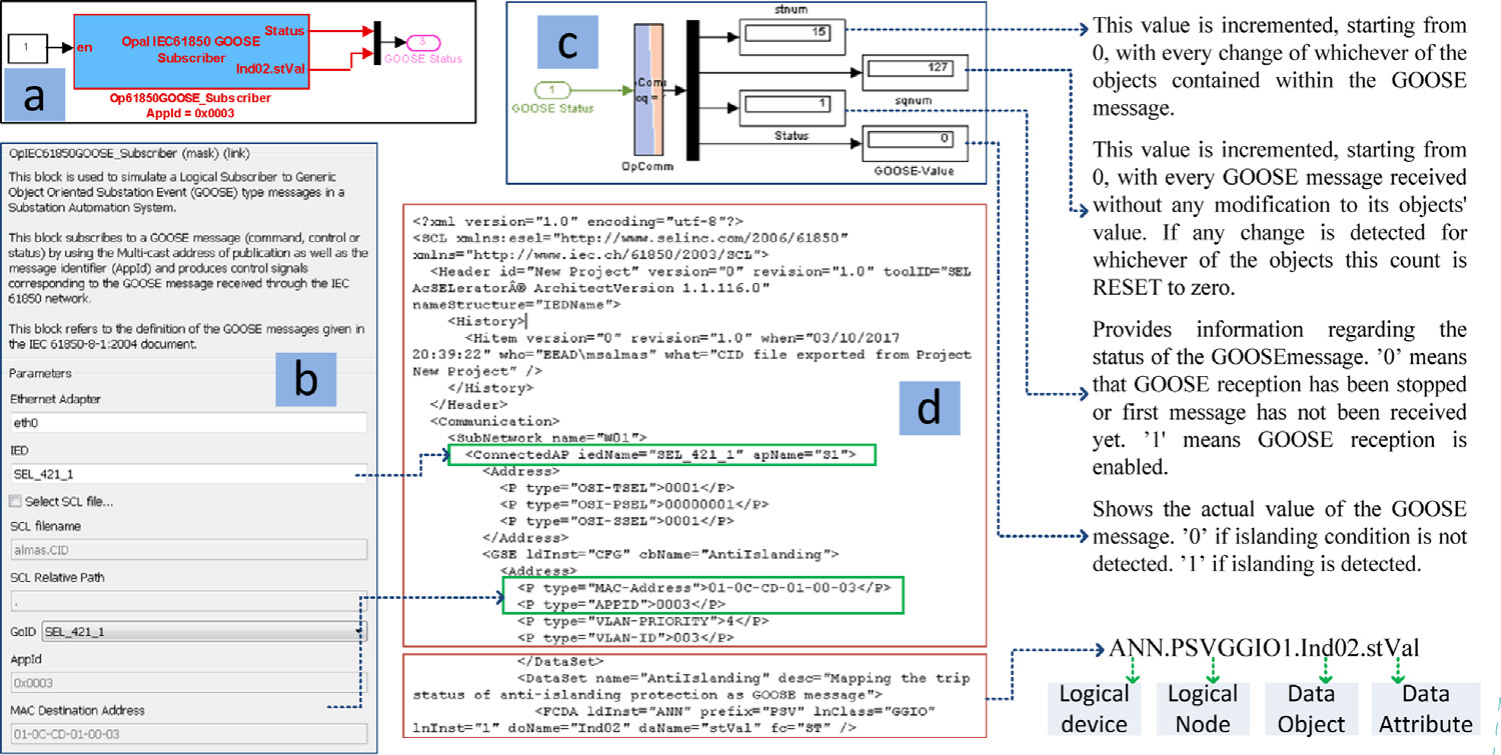
Configuring “GOOSE Subscriber” Block. (a) GOOSE Subscriber block in Simulink enviroment, (b) parameters required to configure the block, (c) description of the outputs of the block, (d) snippet of the CID file of the PMU-B which is used to configure the “GOOSE Subscriber” block.

The four steps of this method (3.1–3.4) are carried out by configuring the PMUs, and then, executing RT-HIL simulations. These configurations are made through the SEL AcSELerator Quickset software interface that allows to access and modify different PMU settings. The important settings required to deploy this method using SEL PMUs is shown in Fig. 7, Fig. 8, Fig. 9 and its description is provided below:

• Step 1: Configuring PMU-A (remote) and PMU-B (local) to compute synchrophasors for voltage phases (A, B, C) and its positive sequence of Bus 2 and Bus 1 respectively. Enabling Port 1 and Port 2 in PMU-A (remote) and PMU-B (local) respectively, to communicate via synchrophasor protocol. Finally PMU-B (local) is configured as a client for PMU-A (remote) to receive remote synchrophasors i.e. voltages of phase A, B, C and positive sequence voltage of Bus-2. These steps are shown in Fig. 7.
• Step 2–3: PMU-B computes the synchrophasors of Bus 1 (Fig. 8a), receives remote synchrophasors of Bus 2 computed by PMU-A through Port 2, and parses them (Fig. 8b and Fig. 8c). These remote synchrophasors are mapped to local variables as shown in Fig. 8d. To clarify, note that in Fig. 8d, the first remote synchrophasor has a magnitude of 0.998 kV and corresponding angle of 76.492°, it is mapped to local variable RTCAP01 (remote synchrophasor positive sequence voltage) and RTCAP02 (remote synchrophasor positive sequence voltage phase angle) respectively (Fig. 8d). The local and remote synchrophasors are utilized internally in PMU-B to execute the anti-islanding protection algorithm. This algorithm is deployed using protection logic equations and generates a trip signal for anti-islanding if the difference between local and remote synchrophasors is greater than 15° for 10 cycles.
• Step 4: Important outputs, such as alarms and PMU status provided by the algorithm, are mapped to the physical outputs, front panel LEDs, Human-Machine Interface (HMI) and GOOSE messages of the PMU-B. This provides quick indication of the status of anti-islanding protection scheme and allows the trip signals to be accessed either from the physical outputs (available at the rear panel of the PMU as shown in Fig. 4) or as IEC 61850-8-1 GOOSE messages through the Ethernet port of the PMU-B. This GOOSE message is received by the Opal-RT’s RTS through its Ethernet port. Inside the RTS, this GOOSE message is parsed using “GOOSE Subscriber” block in the Simulink environment. The output of the GOOSE subscriber block is a binary value (‘0’ for normal operation and ‘1’ for trip) which is wired to the circuit breaker CB-1 to disconnect the generator from its local load and thus executing the anti-islanding protection. The important settings of PMU-B required for publishing the GOOSE message is shown in Fig. 9. The data-flow from the publishing of GOOSE message by PMU-B to its reception in RTS and parsing in Simulink environment using “GOOSE Subscriber” block is shown in Fig. 10. The parameters required for configuring this “GOOSE Subscriber” block is shown in Fig. 11(b) and the description of the outputs of this block is shown in Fig. 11(c). The snippet of the Configured IED Description (CID) file of the PMU-B is shown in Fig. 11(d).

## Method results

**Fig. 12:**
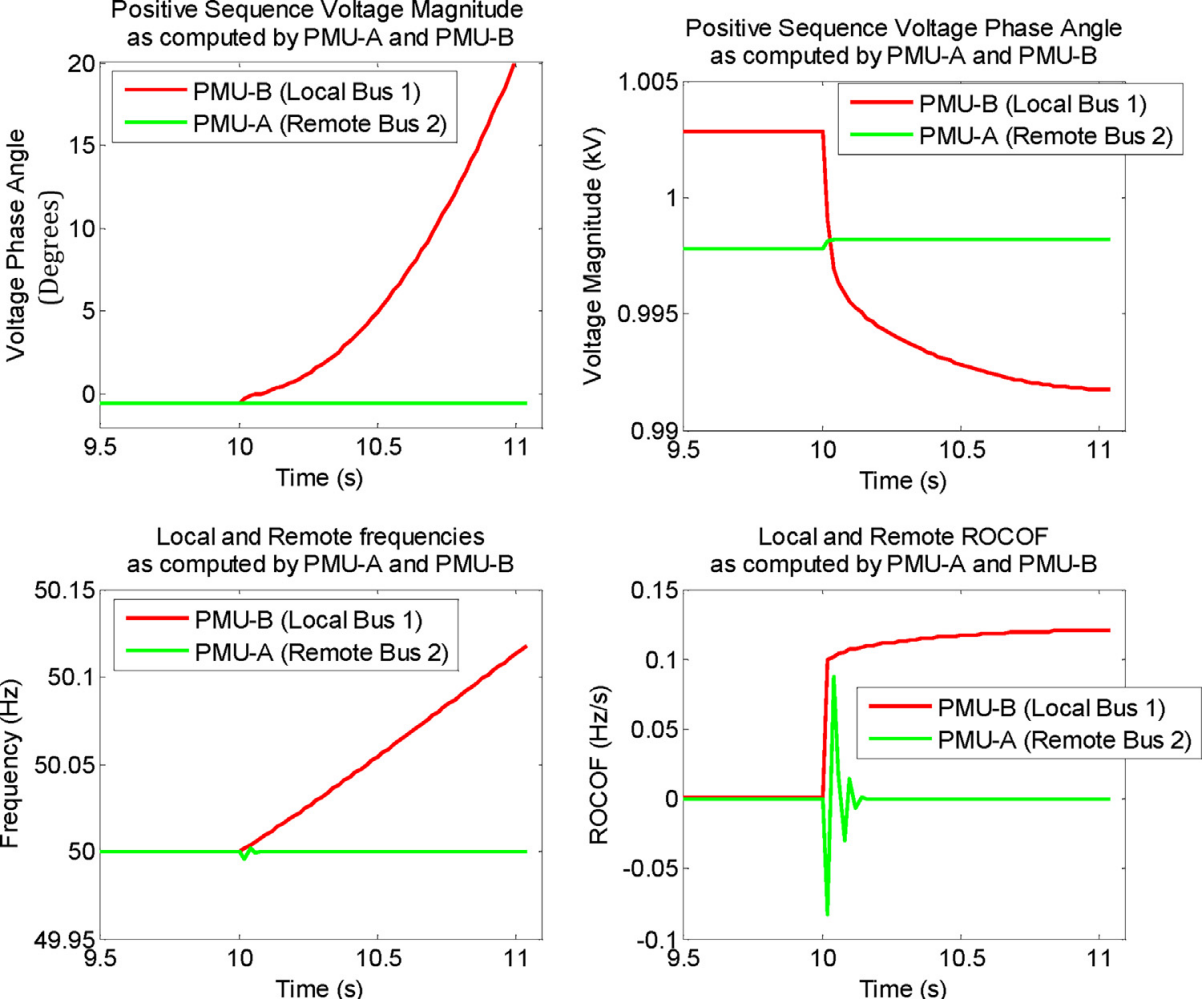
Local and remote synchrophasors as computed by PMU-B (Bus 1) and PMU-A (Bus 2) respectively. Plots show the local and remote synchrophasor positive sequence voltage magnitude, phase angle, frequency and Rata-of-change-of-frequency (ROCOF) measurements.

**Fig. 13:**
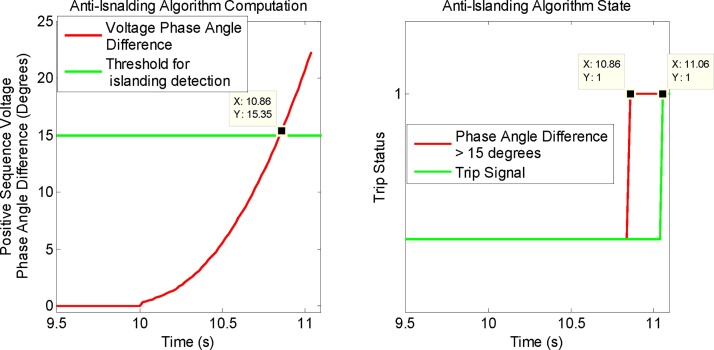
(Left) Computations performed by PMU-B (Client) by utilizing both local and remote synchrophasors. (Right) Trip signal generated by anti-islanding algorithm when voltage phase angle difference exceeds 15° and 10 cycle timer elapses.

**Fig. 14:**
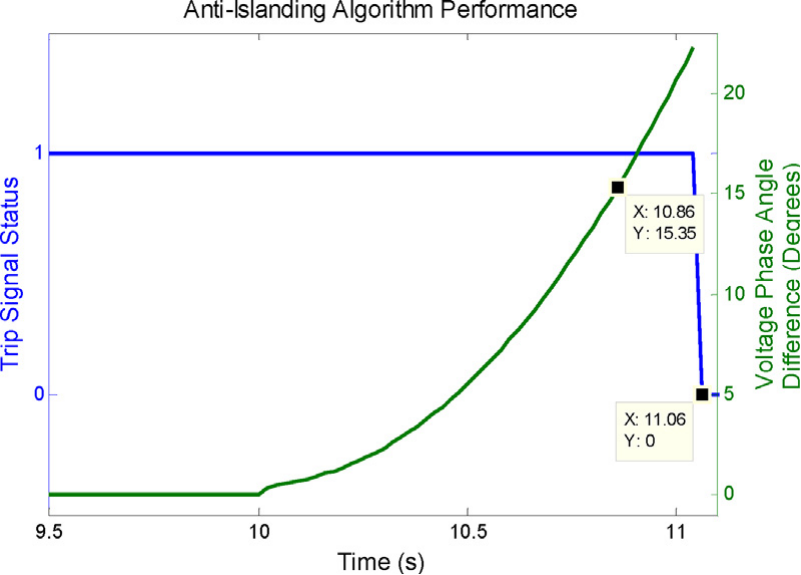
Anti-islanding algorithm performance analysis. For 5% active power mismatch between generator and local load, the anti-islanding algorithm has operated in 1.06 s, which is well within the 2 s limit specified by IEEE Std. 1547-2008.

RT-HIL simulation results of the anti-islanding protection scheme deployed using the proposed method are shown in Fig. 12, Fig. 13, Fig. 14. Local (Bus 1) and remote (Bus 2) synchrophasor positive sequence voltage, frequency and ROCOF computed by PMU-B and PMU-A respectively are shown in Fig. 12. These results correspond to a scenario where active power mismatch between generator and the local load is 5%. At t = 10 s, the circuit breakers CB-2a and CB-2b opens to isolate generator and its local load from the rest of the gird. Thus it results in an island condition.

As soon as the island is formed, the 5% active power mismatch between generator and its local load in the islanded region results in an increase in frequency and corresponding changes in phase angle and ROCOF as computed by PMU-B (local PMU) and shown in Fig. 12. As PMU-A (remote PMU) is connected to Bus-2 that is a part of the strong network, therefore the changes in its system state are minimal because of the limited impact of the island’s formation.

The islanding algorithm executing in PMU-B utilizes both local and remote synchrophasor positive sequence voltage phase angles to compute their phase angle difference and to check if this difference exceeds the threshold of 15°. This computation by the algorithm is shown in Fig. 13. When the phase angle difference violates the 15° threshold, the algorithm starts the timer PCT01 (Fig. 8 Right). When the timer elapses after 10 cycles (200 ms) and the phase angle difference still exceeds 15°, the algorithm issues a trip signal as shown in Fig. 13.

The performance analysis of the scheme is shown in Fig. 14. The island is formed at 10 s because of opening of CB-2a and CB-2b. The phase angle difference exceeds 15° at 10.86 s. At this time, the timer starts and it elapses after 10 cycles (200 ms), i.e. at t = 11.06 s, when the trip signal is generated which is mapped to the physical output of the PMU-B and to its front panel LEDs. The total trip time of this anti-islanding scheme for this scenario is the difference between the time when the trip signal is generated (t = 11.06 s) and the time when the island is formed (t = 10 s). So the total operating time of this scheme is t = 1.06 s which is well within the 2 s limit specified by IEEE Std. 1547-2008 [12].

**Fig. 15:**
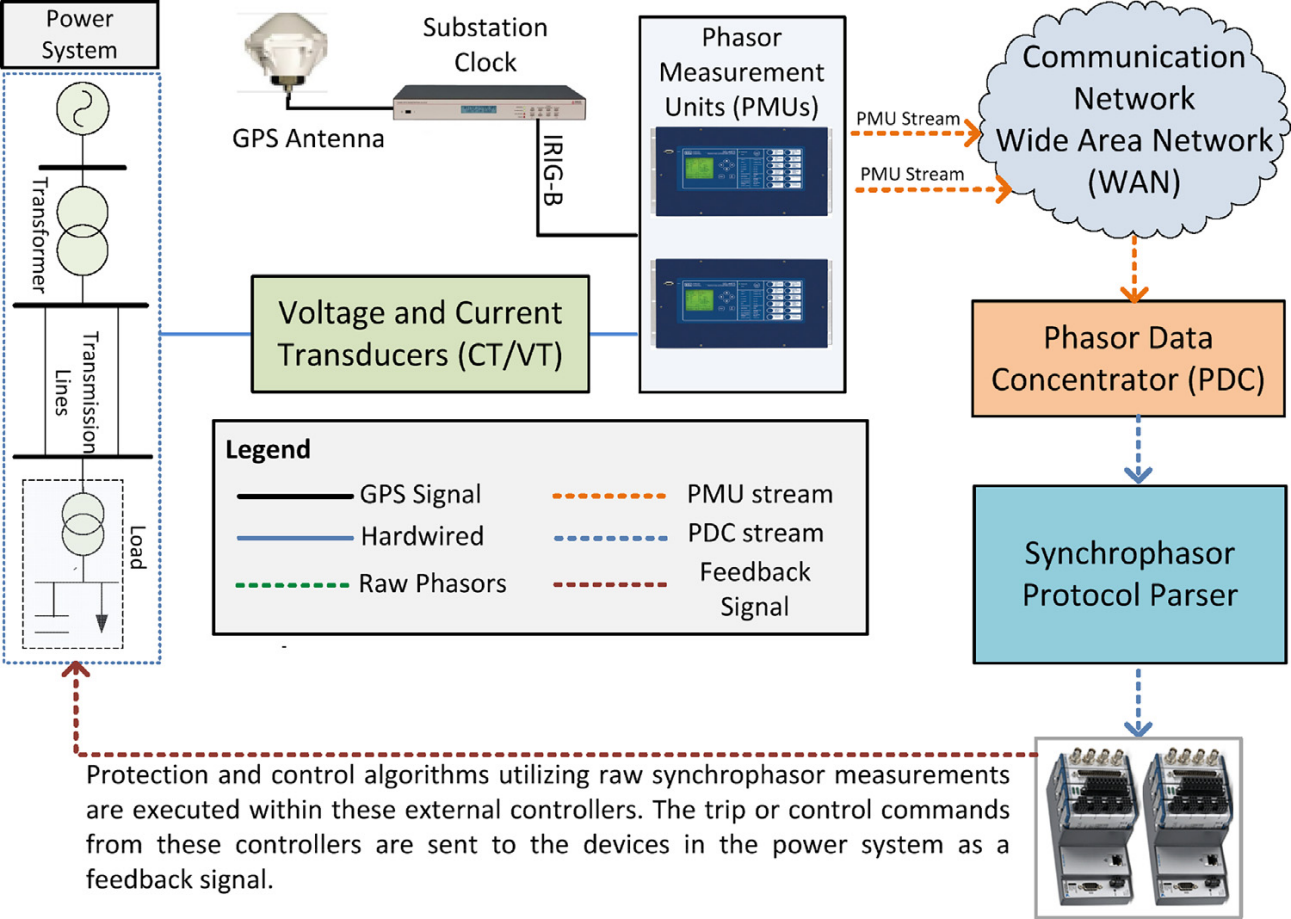
Typical architecture of a synchrophasor-based protection and control application.

The potential of the proposed method is further elaborated by performing a comparative analysis. This is achieved by deploying the same anti-islanding protection scheme using typical synchrophasor architecture as shown in Fig. 15. The test setup consists of (i) two PMUs where PMU-A is connected to the remote bus (Bus 2) while PMU-B is connected to the local bus (Bus 1), (ii) a Phasor Data Concentrator which concentrates the two synchrophasor streams, (iii) synchrophasor protocol parser executing in a workstation which unwraps the synchrophasor protocol and provides raw measurements to the external controller, (iv) external hardware controller executing the anti-islanding protection algorithm using the parsed synchrophasor data, and (v) hard-wiring the trip signal generated from the external controller to the RTS to open circuit breaker CB-1.

**Fig. 16:**
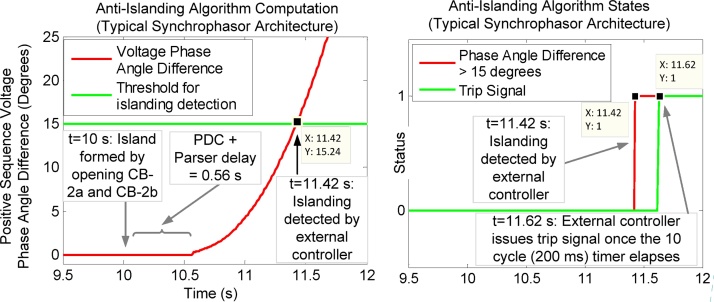
(Left) Computations performed by external controller to calculate positive sequence voltage phase angle difference from the parsed synchrophasor data obtained from PMU-A (remote) and PMU-B (local). (Right) Trip signal generated by anti-islanding algorithm being executed in the external controller. This trip signal is generated when voltage phase angle difference exceeds 15° and 10 cycle timer elapses.

The same scenario of 5% active power mismatch between generator and the local load is simulated. At t = 10 s, the circuit breakers CB-2a and CB-2b opens to isolate generator and its local load from the rest of the gird. Thus it results in an island condition. The performance analysis of this anti-islanding scheme when deployed using typical synchrophasor architecture is shown in Fig. 16.

When the island is formed at t = 10 s, the positive sequence voltage phase angle difference as computed by the anti-islanding algorithm (executing in the external controller) increases. The external controller generates a trip command when this phase angle difference exceeds 15 degrees and remains above this value for 10 cycles (200 ms). This trip signal is generated at t = 11.62 s, at which time the circuit breaker CB-1 opens to disconnect the generator from the isolated grid (island). So the total operating time of this scheme (when using typical synchrophasor architecture) is t = 1.62 s. When compared to the operation time of the same scheme using proposed (direct communication between PMUs), this difference is 0.56 s. This increase in operation time is due to the processing time of the intermediate PDC, protocol parser and the external hardware controller, which are not incurred in the proposed method where a direct communication is established between the PMUs. It is worth mentioning that in typical synchrophasor architecture, the intermediate PDC and the protocol parser are software executing in workstations with non-real-time operating system (RTOS).

This additional delay in operating time of the anti-islanding protection scheme may violate the criteria (total operating time of 2 s) specified by IEEE Std. 1547-2008 [12]. Furthermore, the additional delay can result in false activation of the back-up protections which may result in cascading failures leading to blackout. In case of synchrophasor-assisted control applications such as power oscillation damping, this delay in the feedback loop can degrade the performance of oscillation damping controller or incorporate negative damping into the system [20].

## Conclusions

A method for establishing direct communication between Phasor Measurement Units (PMUs) for deploying custom Wide-Area Protection and Control (WAP&C) algorithms was presented. The method configures one PMU as a client for the other PMUs to receive synchrophasors directly and utilize them internally in custom algorithms. WAP&C algorithms are deployed using protection logic equations. The method allows for fast prototyping of real-time wide-area measurement based protection and control algorithms without requiring any additional hardware controller or intermediate data processing and protocol parsing applications. Therefore, the method not only reduces testing and deployment costs of such applications, but also reduces the overall communication latencies introduced due to the intermediate applications.

Although the use of protection logic equations limits the type of control and protection applications that can be deployed, the use of this approach in the proposed method aims to reduce the WAP&C deployment and application cost & effort, which is otherwise prohibitively high and requires deep know-how from engineers. In contrast, this proposed method allows a lower cost and effort means to exploit PMU measurements in an environment familiar to protection engineers.

The method was tested in real-time using commercial PMUs in Real-Time Hardware-in-the-Loop (RT-HIL) setup. For illustration and validation purposes, a wide-area measurement based anti-islanding algorithm was deployed in one of the PMUs and its performance was assessed. This was achieved by executing a power system model in real-time using Opal-RT’s eMEGAsim Real-Time Simulator (RTS), feeding the three phase voltages and currents of local and remote buses to commercial PMUs and establishing a direct communication between them. All the important steps of the method along with the necessary configurations required in the commercial PMUs, for WAP&C deployment are also presented. The performance analysis of the proposed method concludes that the remote synchrophasors from a commercial PMU were received successfully in the local PMU and the local PMU executed the anti-islanding algorithm utilizing both local and remote synchrophasors precisely to generate trips, activate alarms and transferring trip signal through its physical output/GOOSE message to isolate the generator and accomplishing anti-islanding protection. The proposed method is generic and can be used to test, validate and deploy any WAP&C application that utilizes measurements from two or more PMUs. The utilization of this method for deploying three different types of anti-islanding schemes and synchrophasor-based synchronization scheme is reported in [1] and [2] respectively.

## Additional information

Synchrophasors from Phasor Measurement Units (PMUs) are considered as one of the enablers of the evolving smart grid [14]. PMUs provide accurate and time-synchronized measurements of electrical quantities including voltage and current phasors, at a reporting rate as high as 50/60 msgs per second. PMUs stream out these synchrophasors using IEEE C37.118.2 protocol [5]. Synchrophasors from multiple remote measurement points in the grid not only give more observability for power system monitoring, but also provide fast and near real-time detection for certain power system phenomena, such as islanding detection and power oscillation damping [15], [16], [17], [18], [19].

The typical architecture of a synchrophasor-based Wide Area Protection and Control (WAP&C) application is shown in Fig. 15. Three phase voltages and currents of the desired buses in the power system are fed to the PMUs through voltage and current transformers, respectively. These PMUs compute voltage and current phasors [21] and time-tag them using a time synchronization signal from a substation clock [22] that receives timing signals from satellites through its GPS antenna. Once the synchrophasors are computed, PMUs stream them out through their Ethernet port using the IEEE C37.118.2 protocol [5]. Multiple PMU streams are received in a Phasor Data Concentrator (PDC) [23] through a communication network. PDC sorts all the incoming synchrophasor streams, time aligns them and concentrates them into a single output stream. The PDC may also provide data archiving services. In order to utilize these synchrophasors for developing WAP&C applications, the concentrated synchrophasor streams from the PDC need to be parsed. This is achieved through a synchrophasor protocol parser that unwraps the synchrophasor stream and provides access to the raw-numerical values of the synchrophasors available within the stream [24]. These raw synchrophasors are utilized in external controllers executing WAP&C algorithms. The output of these controllers in the form of trip or control command signals is configured as a feedback signal to power system components to provide power system protection and control functionalities [25].

As shown in Fig. 15, a realization of a WAP&C application requires some essential intermediate data processors in the form of a PDC and a protocol parser. These PDC and protocol parsers are, in principal, software executing on a non-real-time operating system (non-RTOS). Therefore they introduce non deterministic delays in the protection and control feedback loop [26]. Furthermore, the deployment of WP&C algorithm requires an external controller that can execute these algorithms as fast as the synchrophasors update rate to ensure real-time performance. Therefore, the present deployments of WAP&C applications is complex due to the requirement of external hardware controllers and that may also be subjected to non-deterministic communication delays due to PDC and protocol parsers.
